# Endoplasmic reticulum stress-induced release and binding of calreticulin from human ovarian cancer cells

**DOI:** 10.1007/s00262-021-03072-6

**Published:** 2021-11-20

**Authors:** Trefa M. Abdullah, Jacqueline Whatmore, Edwin Bremer, Rimantas Slibinskas, Marek Michalak, Paul Eggleton

**Affiliations:** 1 Institute of Biomedical and Clinical Science, University of Exeter Medical School, Exeter, UK; 2 Department of Experimental Hematology, Section Immunohematology, Cancer Research Center Groningen (CRCG), University of Groningen, University Medical Center Groningen, Groningen, The Netherlands; 3 Department of Eukaryote Gene Engineering, Institute of Biotechnology, Life Sciences Center, Vilnius University, Sauletekio ave. 7, 10257 Vilnius, Lithuania; 4 Department of Biochemistry, University of Alberta, Edmonton, AB T6G 2H7 Canada; 5 College of Pharmacy, Department Biochemistry and Clinical Chemistry, University of Sulaimani, Iraqi Kurdistan Region, Sulaimani, Iraq; 6 Revolo Biotherapeutics, New Orleans, LA 70130 USA

**Keywords:** Thapsigargin, Doxorubicin, Tauroursodeoxycholic acid (TUDCA)

## Abstract

**Background:**

Calreticulin (CRT) is an endoplasmic reticulum (ER) chaperone, but can appear surface bound on cancers cells, including ovarian cancers (OC). We investigated at what stage of cell viability, CRT appeared associated with surface of human OC cells. CRT on pre-apoptotic tumour cells is thought to initiate their eradication via a process termed immunogenic cell death (ICD).

**Methods:**

We treated OC cells with the chemotherapeutic—doxorubicin (DX) known to induce translocation of CRT to some tumour cell surfaces, with and without the ER stressor—thapsigargin (TG)—and/or an ER stress inhibitor—TUDCA. We monitored translocation/release of CRT in pre-apoptotic cells by flow cytometry, immunoblotting and ELISA. We investigated the difference in binding of FITC-CRT to pre-apoptotic, apoptotic and necrotic cells and the ability of extracellular CRT to generate immature dendritic cells from THP-1 monocytes.

**Results:**

Dx-treatment increased endogenously released CRT and extracellular FITC_CRT binding to human pre-apoptotic OC cells. DX and TG also promoted cell death in OC cells which also increased CRT release. These cellular responses were significantly inhibited by TUDCA, suggesting that ER stress is partially responsible for the changes in CRT cellular distribution. Extracellular CRT induces maturation of THP-1 towards a imDC phenotype, an important component of ICD.

**Conclusion:**

Collectively, these cellular responses suggest that ER stress is partially responsible for the changes in CRT cellular distribution. ER-stress regulates in part the release and binding of CRT to human OC cells where it may play a role in ICD.

**Supplementary Information:**

The online version contains supplementary material available at 10.1007/s00262-021-03072-6.

## Introduction

Ovarian carcinomas account for five percent of the total cancers in women and a quarter of the malignancies of the female genital tract. However, it is the most common cause of death among women who develop cancers of gynaecologic origin [1]. Ovarian cancer once diagnosed is initially treatable, as the majority will respond temporarily to surgery and cytotoxic agents. The disease, however, frequently persists and recurs, having the highest fatality-to-case ratio of all the gynaecologic cancers [2]. Despite these discouraging statistics, improvement in 5-year survival has occurred steadily over the last three decades with more aggressive surgical management and the development of more effective chemotherapy [3]. The discovery that the translocation of the ER chaperone—calreticulin (CRT) to the cell surface of some tumour cells, where it acts as a ‘eat-me’ molecule [4], may enhance the immunogenicity of early apoptotic cancer cells by triggering both innate immunity (phagocytosis and a specific adaptive immune response termed immunogenic cell death (ICD) [5], has drawn attention to CRT for aiding cancer cell recognition by the immune system in recent years [6–9]. It has been proposed that the translocation of endogenous CRT to the cell surface of pre-apoptotic tumour cells enhances their recognitions and engulfment by dendritic cells [10, 11].

CRT is a highly conserved endoplasmic reticulum (ER) chaperone protein that also displays extracellular multifunctional properties in various cellular processes [12]. It was first identified as a Ca^2+^-binding protein in 1974 [13]. Accumulating evidence points to the hypothesis that CRT has an impact on the development of different cancers and the effect of CRT on tumour formation and progression may depend on cell type and clinical stages. [14]. We and others have highlighted various roles of extracellular CRT in immunity [15–17] and have observed an anti-tumour role for CRT [18]. CRT may represent a phagocytic signal on tumour cells recognized by dendritic cells, natural killer cells and other cells of both innate and adaptive immunity [19, 20].

Interestingly, apoptotic cell death induced by treatment with the chemotherapeutic agent doxorubicin (DX) has been reportedly found to induce early apoptosis characterized by release of CRT in early (pre)apoptotic stages in some cell types. In these settings, CRT is thought to act as an ‘eat-me’ signal to immature dendritic cells and initiate immunogenic cell death (ICD) [8]. However, DX has been reported to exert its anti-neoplastic effect by intercalation into DNA and is known to cause DNA damage, oxidative stress and cell death [21]. Moreover, there is recent evidence to support DX inducing ER stress in a number of cell types [22, 23]. In addition, thapsigargin (TG) is a well-characterized ER calcium pump inhibitor and ER stressor [24]. Recent studies at the messenger RNA level suggest lack of CRT message in ovarian cancer cells correlated negatively with cytotoxic T-cell infiltration into tumour sites in ovarian cancer patients and negatively affects survival [25]. Based on these reports, we have investigated the cellular stress conditions that influence the ability of CRT to be released from ovarian cancer cells or bind to these cells, to further understand the mechanisms that influence CRT release from the ER onto plasma membrane and/or extracellular space prior to promoting tumour immunity.

## Materials and methods

### Reagents and cell lines

Cell culture and treatment OVcar3, SKov3 epithelial ovarian cancer cells were maintained in RPMI 1640 (Lonza) supplemented with 10% v/v foetal bovine serum, 50 µM gentamycin sulphate and 2 mM L-glutamine (Lonza). Cells were routinely incubated at 37 °C in 5% CO2-95% air atmosphere and passaged twice every 6 days. Ovarian cancer cells (1×10^5^ cells) were plated in 6-well plates for use in flow cytometry analysis. When immunohistochemistry was utilized, cells were cultures in wells containing sterile cover slips to 80% confluence. The following day, cells were treated with varying concentrations of doxorubicin—DX (Sigma) alone (2.5, 6.3, 12.5, 25 µM). Separately, cells were treated with varying concentrations of thapsigargin -TG (0.2, 0.5 µM) with and without 2.5 µM DX for 16 h. All cells were then exposed to 2 µg/ml (final concentration) FITC-CRT for 30 min and washed once in sterile PBS. Next, for flow cytometry analysis, cells were removed from wells of plates by trypsinizing them with 0.4 mls of trypsin—EDTA—0.05% at 37 °C for 5 min before adding 1 ml of DPBS (Dulbecco’s Phosphate Buffered Saline—Fisher) without calcium, magnesium to neutralize the trypsin. Then, the cells were centrifuged at 1500 rpm for 5 min to obtain a pellet. The DPBS was removed and the cells resuspended in 1 ml of fresh DPBS.

### Generation of immature dendritic cells from THP-1 monocytic cells

THP-1 cells (ATCC TIB202) were harvested by centrifugation, resuspended in culture medium supplemented with 10% (v/v) FCS at a concentration of 2 × 10^5^ cells/ml and transferred in a final volume of 14 ml into 75 cm^2^ tissue culture flasks. To induce differentiation into imDCs, IL-4 (500 IU/ml) and GM-CSF (800 IU/ml) were added. Cells were cultured for 7 days with change of media every 2 days by substituting half of the media with fresh media containing fresh cytokines. Maturation of DCs was assessed by surface expression of markers of maturation, i.e., CD14, CD83, CD11c, CD80, HLA-DR and CD86 by flow cytometry (Table S1). Lipopolysaccharide (LPS) is known to activate antigen-presenting cells. Thus, LPS was added at 1 μg/ml as a positive control to induce maturation of imDCs from THP-1 cells using the protocol of Sim and coworkers [26]. Yeast-derived—LPS -free extracellular CRT alone (0.5–2.5 µg/ml) was used to see if it can also induce DC maturation.

### Human CRT expression and fluorescein isothiocyanate (FITC) conjugation

Full-length human CRT precursor (Gen- Bank Acc. no. M84739) was PCR amplified from a human liver cDNA library and cloned into the yeast expression vector pPIC3.5K (Invitrogen) and transformed into P. pastoris strain GS115 (his4). G418-resistant transformants were selected for protein expression and purification of recombinant CRT, as previously described [27]. Recombinant CRT was FITC conjugated using our previously published method [28]. Briefly, a 54 µM solution of CRT, comprising of 0.5 mg in 200 µl of conjugation buffer (0.1 M sodium carbonate buffer—pH 9.0), was incubated with 50 µl of 0.5 mg/ml (128 µM) FITC solution in conjugation buffer for 2 hours at room temperature in the dark. The resulting FITC-CRT was separated from free FITC and buffer exchanged into PBS (pH 7.4) through a 5 ml capacity desalting G-25 Sephadex column attached to an AKTA FPLC purifier (GE Healthcare) and the FITC: protein ratio calculated. Commercially prepared human FITC-IgG (Sigma—F9636) was used as a control cell binder.

### Immunoblots

To study cellular and extracellular release of CRT, confluent cells (1 × 10^5^ cells/well) from 6-well plates after various treatments for 16 hours were trypsinized to obtain a pellet and immediately solubilized in 200 μl ice-cold lysis buffer (1% w/v SDS, 1% v/v Triton × 100, 50 mM Tris (pH 7.5), 150 mM NaCl and 1 × strength protease cocktail inhibitors (Thermofisher prod no. 88266). The lysates were centrifuged at 340xg at 4 °C for 20 min before collecting the cell free lysates. Protein concentrations were determined by nanodrop spectroscopy and BCA protein assay kit [29]. Samples were adjusted to the same protein concentration. β-actin was quantified as a loading control. Then, 20 μl aliquots in sample buffer was run on Mini-PROTEAN® 10 % SDS-PAGE TGX 10-well gels (Bio-Rad). Gels were transferred onto nitrocellulose using a Trans-blot® Turbo™ blotting system (Bio-Rad). Blots were probed with 1:1000 dilution of a 1 μg/ml rabbit anti–human-CRT (Thermofisher A3-900), washed 3x in PBS-0.2% Tween-20 and once in PBS and then incubated at RT for 1 h with a 1:15000 dilution of IRDye 800CW goat anti-rabbit IgG (Licor 926-32211). Blots were washed twice in PBS- 0.2% v/v Tween-20 and once in PBS alone and were analysed on an Odyssey CLx Imager. Supernatants of 1 × 10^5^ cells incubated in RPMI medium with serum were recovered at 0 and 16 h after various treatments. Cell supernatants were isolated by centrifugation at 320 × g for 5 min for further analysis, and an equal volume of trichloroacetic acid (50% w/v in water) was added to each supernatant for one night at 4 °C to precipitate contained proteins. Samples were then assessed for their CRT content by western blotting.

### Flow cytometry

Endogenous CRT expression on the surface of non-permeabilized cells was determined by flow cytometry (Guava easyCyte™ Flow cytometer) using Incyte software 3.11. Following pharmacological treatment, cells were washed and suspended in 1 ml PBS containing 0.5% v/v BSA and immediately incubated with rabbit anti-human-CRT Ab (1:200) for 1 h, followed by further washing x 3 and then stained with a 1:2000 dilution of a secondary antibody—goat anti-rabbit IgG H&L (Alexa Fluor® 488) (ab150077) in the dark for 30 min. The adherent cells were then washed three times in PBS and trypsinized to allow the cells to be placed in suspension. Aliquots of cells were then placed in 1 ml Annexin V binding buffer (Biolegend—PN-422201) and 100 μl aliquots of cells co-stained with 5 μl Alexa Fluor® 647 Annexin V ((Biolegend—640912) to monitor apoptosis and 10 μl propidium iodide-Yellow (Sigma-81845) for 15 min in the dark to assess necrosis (Fig. S1). Finally, the volume was adjusted to 500 μl with 1 × binding buffer and analysed by flow cytometry immediately without fixation. To assess exogenous FITC-CRT binding to cells, the same protocol as described above was employed, except that cells were incubated with a 1:500 dilution of 2 μg/ml FITC-CRT or control FITC-IgG (Fig. S2) for 30 min in the dark, then washed three times and monitored for apoptosis and necrosis as above.

### ELISA assays

CRT released in to 1 ml of culture media from ~ 1 × 10^5^ cells treated with DX and/or TG with or without TUDCA was measured by ELISA [30]. Briefly, 153 μl of cell-free supernatant was placed into wells of a 96-well ELISA plate with 17 μl of 10 × carbonate buffer, pH 9.6. The protein supernatants were left to bind at 4 °C overnight. The plates were then washed four times with phosphate-buffered saline (PBS) containing 0.1% v/v Tween 20 (PBST). Remaining binding sites were blocked with 5% v/v BSA in PBST at 37 °C for 30 min. Wells were washed a further four times with PBST. Next 100 μl of 1:2000 dilution of rabbit anti-human CRT antibody (Thermofisher PA3-900) diluted in PBST was added to each well for 2 h in 37 °C. The wells were washed again as described above, and a 100 μl of 1:2000 dilution of secondary anti-rabbit HRP-conjugated antibody was added to the wells and the plate incubated 1 h in 37 °C. The reaction was developed for 15 min at RT in the dark and was terminated by adding 50 μl 2N H_2_SO_4_ to each well. The optical density at 450 nm (OD45nm) of each sample was measured on a BMG Labtech FLUROstar™ plate reader. A CRT standard curve was generated from known concentrations of CRT (Fig. S3).

### Immunofluorescence and image analysis

Cells grown on sterile cover slips in 6-well plates for 24 h and treated with DX and/or TG for an additional 16 h before being washed with warm PBS and fixed with 4% w/v paraformaldehyde in PBS for 20 min were blocked in 5% w/v sterile BSA in PBS for 10 min and then incubated with (a) antibodies to detect endogenous CRT released from cells that binds to the cell surface, or (b) cells were incubated with exogenous CRT and then examined by immunofluorescence. Briefly, adherent cells were treated with anti-human CRT (rabbit polyclonal, Thermofisher A3-900; 1:200) for one hour. For endogenous CRT detection, cells were then washed and incubated with Alexa Fluor® 488 conjugated secondary antibody (goat anti-rabbit IgG, Abcam-150077; 1:2000) or goat-anti Rab CY5 –Abcam-6564) for 30 min. Cells were washed again and the coverslip containing cells inverted onto a slide with a drop of ProLong® Gold anti-fade mounting medium with DAPI (Invitrogen). For exogenous CRT binding, cells were incubated for 30 min in the dark at RT. Digital images of cells were captured either on a Leica DM4000 B LED fluorescent microscope or Leica DMi8 TCS SP8 Confocal microscope at × 10, × 20 and × 40 magnification using LAS X digital software.

### Statistical analysis

Results are expressed as mean ± SD. Significant differences between groups were assessed by Student t-test for parametric data. *P* values < 0.5 were considered statistically significant. The statistical analysis was done using Graph-Pad Prism 6.0 software (La Jolla, California, USA).

## Results

### Doxorubicin treatment of cancer cells increases both release of endogenous CRT and binding of exogenous CRT to tumour cells

**Fig. 1:**
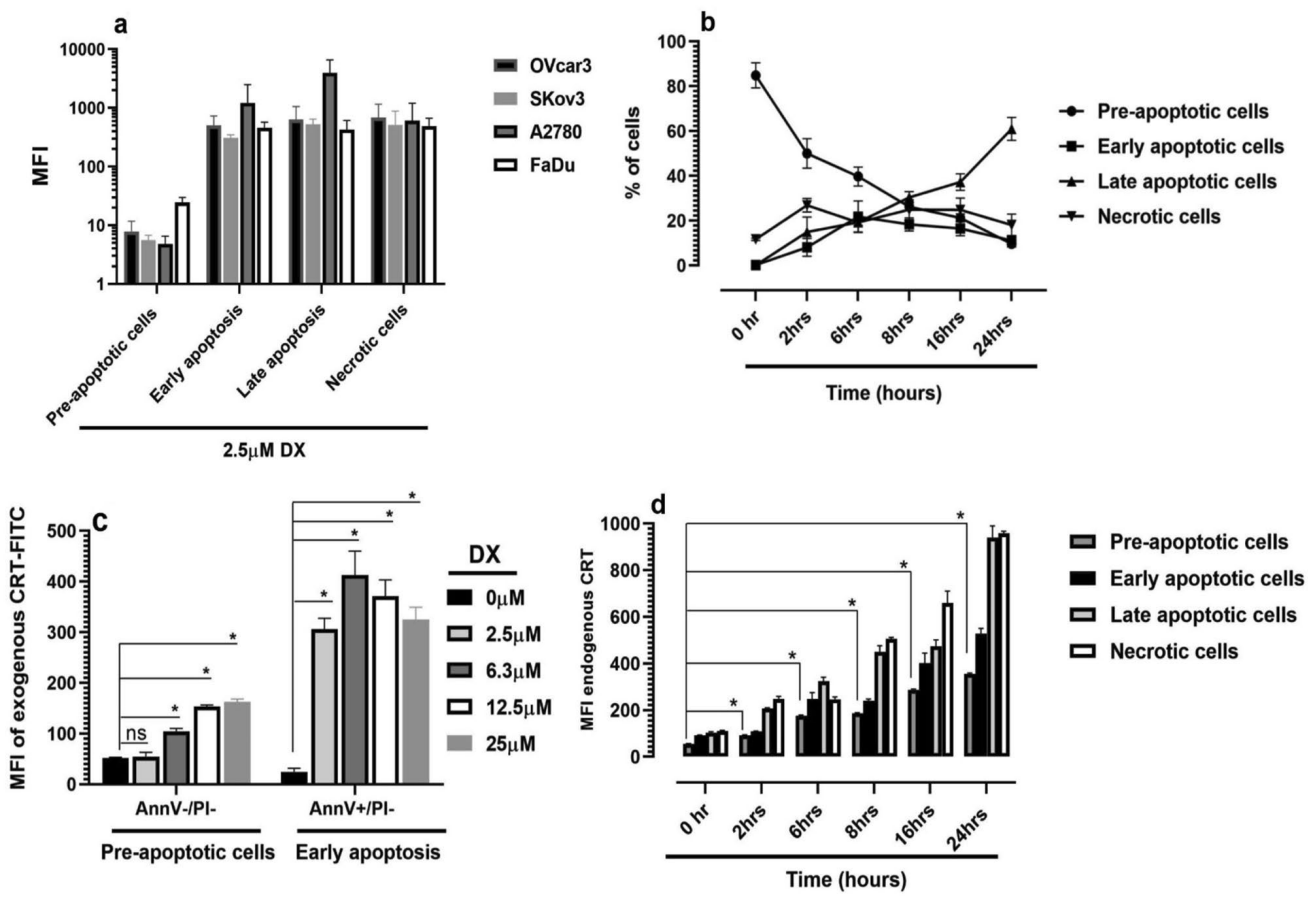
Doxorubicin treatment increases surface presence of endogenously released CRT and extracellular CRT binding on various human cancer cells. **a** Effect of 16 h DX treatment (2.5 µM) on pre-apoptotic, apoptotic and necrotic cancer cells. **b** Mean % + SD of pre-apoptotic, apoptotic and necrotic SKov3 cells pre- and post-DX treatment over 24 h. **c** Mean % + SD of exogenous CRT-FITC binding to pre-apoptotic versus early apoptotic SKov3 cells pre- and post-DX (0–25 µM) treatment for 16 h. *P < 0.05 for no treatment versus Dx treatments. **d** Time course of endogenous CRT (Mean + SD) detected associated with SKov3 cells in various states of cell death, pre- and post-2.5 µM DX treatment; * represents *P* < 0.03 for pre-apoptotic cells untreated versus DX-treatment overtime. Figures **a**–**d** representative of at least ‘three’ independent experiments

Initially we treated four human cancer cell types (Fig. S4) with various concentrations of DX overnight for 16 h. The cell’s apoptotic and necrotic status pre- and post-DX treatment was analysed by flow cytometry using Annexin V (AnnV) and propidium iodide (PI) staining, respectively (Fig. S5). A concentration of 2.5 µM DX consistently increased the detection of endogenous CRT with all four cell types tested (Fig. 1a) as measured by flow cytometry. Treatment of SKov3 cells with this chemotherapeutic predominantly led to an increase in late apoptotic cells and a steady state of early apoptotic and necrotic cells over 24 h (Fig. 1b). To further characterize the nature of extracellular CRT binding to the cell surface, SKov3 cells were treated with a range of DX concentrations and the degree of extracellular FITC-CRT binding to pre-apoptotic or early apoptotic cells was assessed by flow cytometry (Fig. 1c). These cell states were chosen for their AnnV-/PI- and AnnV + /PI- status confirming their intact membrane integrity. This revealed a differential binding of extracellular CRT to DX-treated cells, with early apoptotic SKov3 cells presenting a three–fourfold increase in FITC-CRT binding compared to pre-apoptotic cells. Interestingly, when endogenous release of CRT status was examined pre- and post-2.5 µM DX treatment, there was a statistically significant difference in cell surface CRT on pre-apoptotic cells (AnnV-/PI-) compared to untreated living cells. Not surprisingly, in cells undergoing early/ late apoptosis and necrosis there was greater increase in detection of CRT compared with pre-apoptotic cells, presumably reflecting the gradual ‘increased permeability' of the cell states (Fig. 1d). The higher levels of CRT staining on late apoptotic and necrotic cells could be due to anti-CRT detecting both surface bound and intracellular CRT. Fluorescently labelled secondary antibody alone did not bind to cancer cells (Fig. S6).

**Fig. 2:**
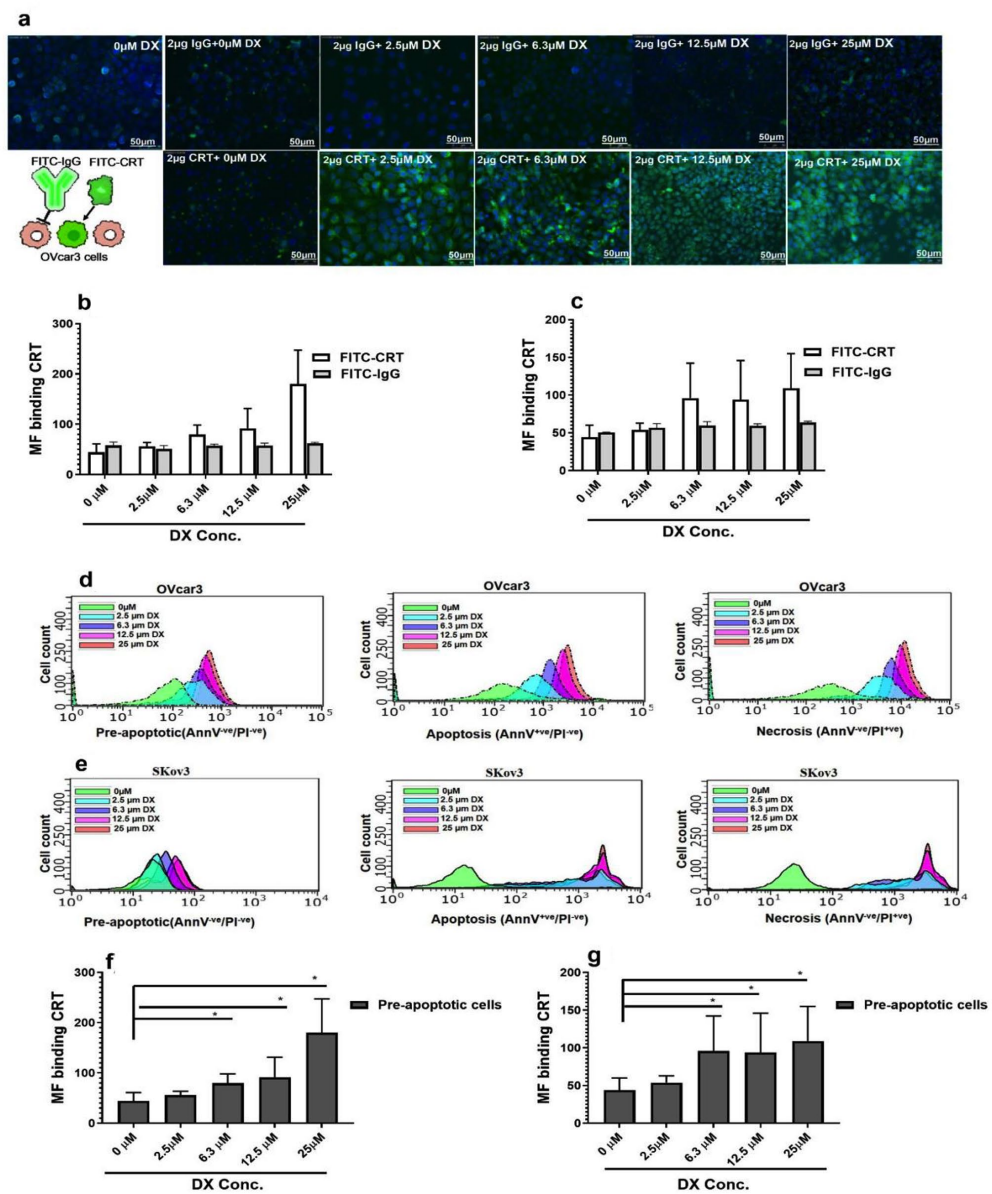
Doxorubicin treatment increases binding of exogenous CRT on non-permeabilized ovarian cancer cells. a Immunofluorescence analysis of FITC-CRT and FITC-IgG binding to DX-treated OVcar3 cells in **a** dose responsive manner. MFI of FITC-CRT and FITC-IgG binding to **b** OVcar3 cells or **c** SKov3 cells in the presence of DX for 16 h. Binding of exogenous FITC-CRT to **d** OVcar3 cells and **e** SKov3 cells in various stages of apoptosis and necrosis. Demonstration of statistically significant binding of exogenous FITC-CRT binding to pre-apoptotic **f** OVcar3 cells and g SKov3 cells (*n* = 3; mean + SD)

Treatment of cancer cells with DX may make them more susceptible to ICD by inducing CRT to the cell surface of pre-apoptotic cells. However, DX is toxic and can promote necrosis leading to cell permeabilization and release of CRT where it may bind to exposed PS or other receptors. We wanted to determine if extracellular CRT binding to human cancer cells was influenced by DX treatment. Both SKov3 and OVcar3 cells were treated with different concentrations of DX (2.5, 6.3, 12.5, or 25 µM) for 16 h, and the cells were exposed to FITC-recombinant CRT (2 µg/ml) or FITC-IgG (control) for 30 min at 37 °C in the dark. After incubation with FITC-CRT or FITC-IgG, the cells were washed to remove unbound excess FITC-protein conjugates. In (Fig. 2a), the binding of FITC-CRT to OVcar3 cells by fluorescent microscopy compared to FITC-IgG is shown. The binding of FITC-CRT and FITC-IgG to OVcar3 and SKov3 cells was analysed by flow cytometry (Fig. 2b, c). Upon treatment with DX, a greater amount of extracellular FITC-CRT bound to both OVcar3 and SKov3 cells compared to DX-untreated cells. As the dose of DX was increased, both cell types were observed to bind more extracellular FITC-CRT (Fig. 2d, e). We were interested to know if extracellular FITC-CRT could bind to pre-apoptotic cells. To investigate this possibility, using flow cytometry, we gated the (pre-apoptotic—AnnV^−ve^/PI^−ve^) Ovcar3 and SKov3 cells (Fig. S1) treated with increasing doses of DX. It was evident pre-apoptotic Ovcar3 (Fig. 2f) and SKov3 (Fig. 2g) cells treated with a range of DX led to a statistically significant increase in binding of exogenous CRT to both types of cells (*P* value < 0.01 in all cases). We are unsure what leads to CRT binding to pre-apoptotic cells, but anthracyclines such as DX are positively charged drugs and are known to interact with negatively charged lipids possibly altering the biophysical features of lipid monolayers [31].

### Treatment of SKov3 cells with thapsigargin or doxorubicin alone or combined increases detection of endogenous CRT

The ER lumen is sensitive to Ca^2+^ changes and ATPase pumps regulate the amount of transport of calcium in and out of the ER. Thapsigargin (TG) inhibits sarcoplasmic/endoplasmic reticulum (SERCA) Ca^2+^−dependent ATPases leading to calcium depletion of the ER stores and activation of unfolded protein response (UPR), an ER stress coping response. Activation of UPR leads to changes in the expression of chaperones, including CRT and an accumulation in unfolded proteins. Under these conditions, CRT translocates to the cell surface [17] possibly by a secretory pathway as cell surface CRT appears can be inhibited by the ER-Golgi protein transporter inhibitor—brefeldin A. Similarly, anthracyclines such as DX are thought to promote externalization of CRT via a secretory pathway [9].

**Fig. 3:**
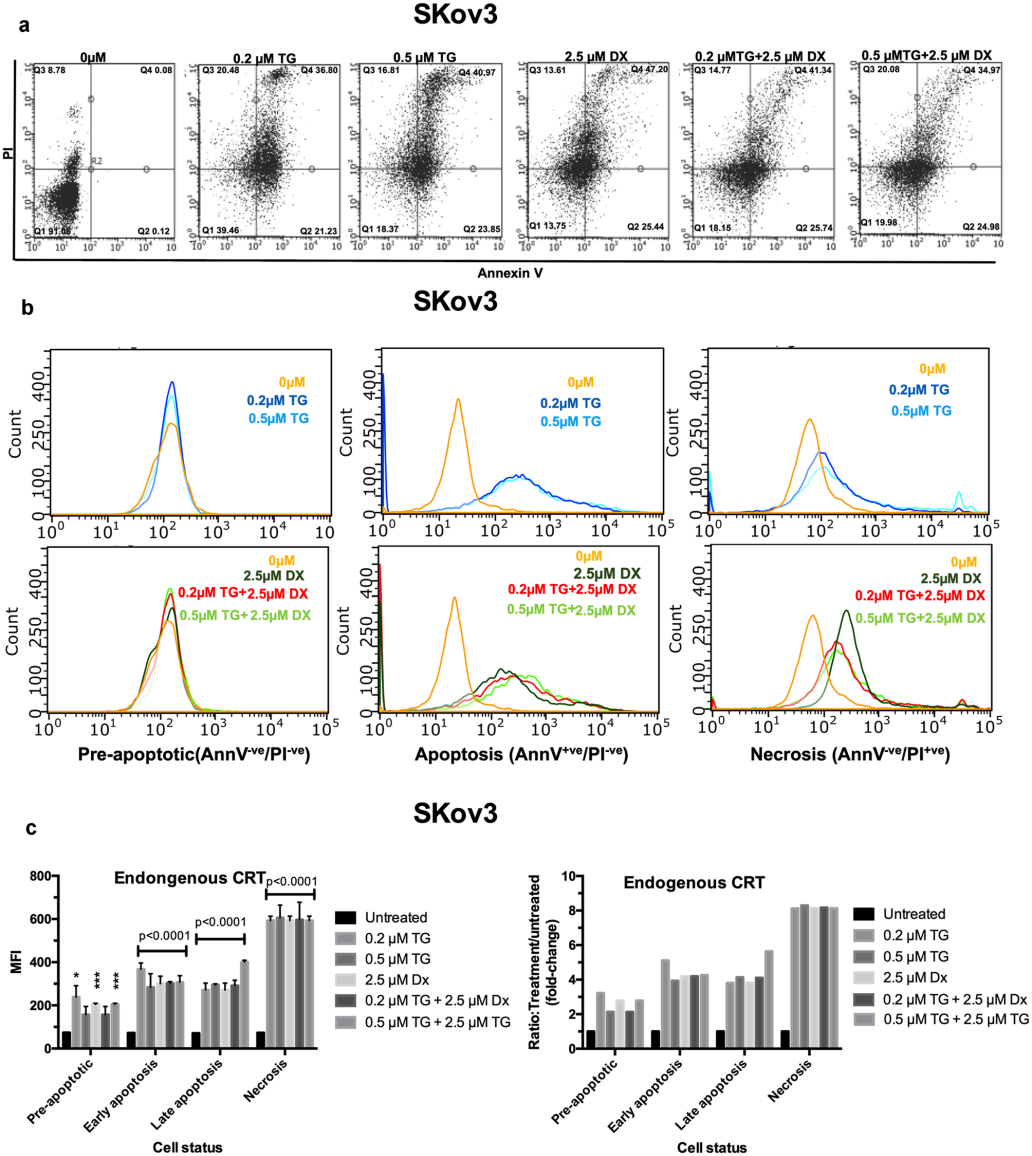
Detection of endogenous CRT in SKov3 cancer cells under various viability and drug-induced stress conditions. **a** Dot plot graphs show the changes in the percentage of viable cells, early apoptotic cells, late apoptotic cells and necrotic cells of SKov3 cancer cells, assessed by flow cytometer with Alexa Fluor® 647 Ann V (apoptosis) and PI (necrosis) probes. Living cells are in the lower left quadrant, necrotic cells permeable to PI only are in the upper left quadrant, and the early apoptotic cells stained by Ann V and unstained by PI in the lower right quadrant. The late apoptotic cells staining for Ann V and PI are shown in the upper right-hand quadrant. **b** Differences in cell surface CRT expression as monitored by changes in mean fluorescent intensity on pre-apoptotic, apoptotic, and necrotic SKov3 cells after treatment with various combinations of DX (2.5 µM), TG (0.2, 0.5 µM) for 16 h. The apoptosis/necrosis status was determined by flow cytometry using AnnV/PI staining before and after treatment with DX (2.5 µM) and/or TG (0.2, 0.5 µM) for 16 h. **c** The MFI of detectable endogenous CRT in SKov3 cells under different viability status and fold increase in CRT surface expression in pre-apoptotic cells exposed to various TG and DX treatments. All experiments *n* = 3 mean + SD; * = *P* < 0.05; *** = *P* < 0.0001)

Initially we investigated the effect of TG, DX treatment alone and combined for 16 h on SKov3 cell viability by AnnV/PI status (Fig. 3a). Both agents promoted increased apoptosis/necrosis. When TG and DX were used together, the number of apoptotic cells increased from ~ 0.12 to 25%, and necrotic cells increased from 9 to 15–20% which positively correlated with detection of CRT (Fig. 3b). We examined the expression of endogenously released or accessible CRT on the surface of pre-apoptotic cells in comparison with apoptotic/necrotic SKov3 cells, pre- and post-16 h treatment with TG (0.2, 0.5 µM), or DX (2.5 µM) or (DX, TG) combined treatment. The amount of CRT on the surface of SKov cells expressed as mean fluorescent intensity (MFI) on pre-apoptotic cells was statistically significantly higher in cells treated with TG/DX alone or combined compared to untreated cells (Fig. 3c-left panel). The increase in cell surface CRT in treated pre-apoptotic cells compared to untreated pre-apoptotic cells represented ~ twofold increase in surface CRT (Fig. 3c-right panel). As the cells began to lose membrane integrity in apoptotic and necrotic cells, the detection of CRT increased and appeared highest in necrotic cells exposed to TG or DX treatments. It was not possible to determine in these latter cells if the CRT being detected by flow cytometry was purely membrane bound and it probably represented detection of both extracellular and intracellular CRT by the anti-human CRT antibody probe.

### Inhibition of ER stress reduced externalization of CRT from DX treated cells

**Fig. 4:**
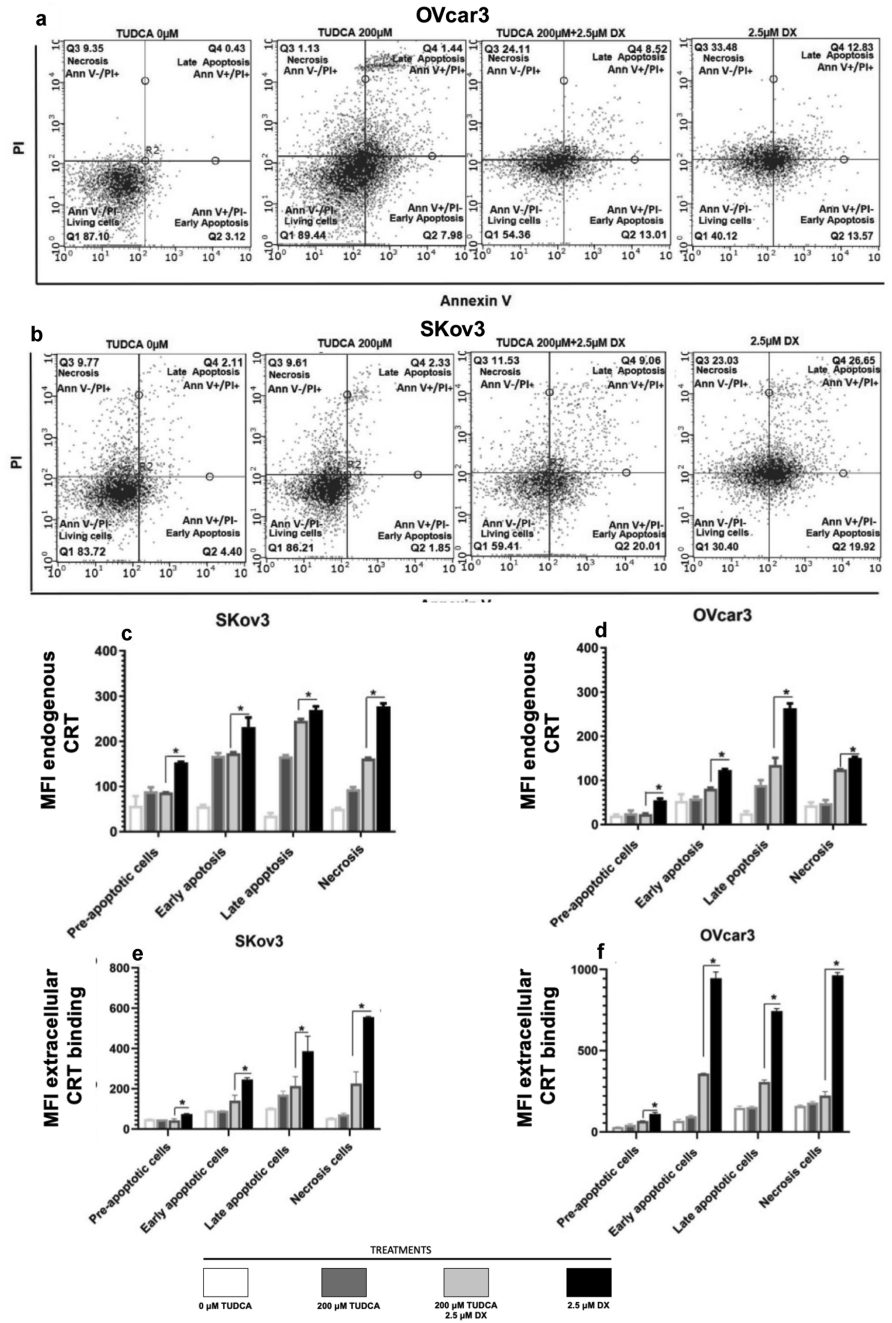
Effect of TUDCA on inhibiting cell death and altering expression of CRT on cell surface of pre-apoptotic cancer cells. Dot plots showing the protective effect of TUDCA on **a** DX induction of OVcar3 cell death and **b** DX induction of SKov3 cell death. MFI of cell surface expression of endogenous CRT release to surface of **c** OVcar3 and **d** SKov3 cells with and without treatment of TUDCA (200 μM) and/or DX (2.5 μM) for 16 h. The effect of TUDCA on extracellular binding of CRT to **e** OVcar3 and f SKOV3 with and without treatment of TUDCA (200 μM) and DX (2.5 μM). The results shown are representative of three independent experiments and analysed by Student t test

We examined the possibility that if DX-induced ER stress is the cause of increased CRT release from pre-apoptotic and other apoptotic/necrotic states, one would expect the ER-stress inhibitor tauroursodeoxycholic acid (TUDCA) would reduce CRT surface exposure. TUDCA is a bile acid, a taurine conjugate of ursodeoxycholic acid and proteostasis promoter [32] known to reduce UPR, an ER stress response, and to stabilize mitochondria [33]. Flow cytometric analysis of PI and Annexin V status indicated that TUDCA blocked the DX-mediated apoptosis and necrosis associated with DX treatment in both OVcar3 (Fig. 4a) and SKov3 cells (Fig. 4b) and confirmed the protective effect of TUDCA on cell death. Interestingly, TUDCA exposure of DX-treated SKov3 (Fig. 4c) and OVcar3 cells (Fig. 4d) led to significantly reduced detection of endogenous CRT in all cell states. In particular, there was also a small but significant decrease in cell surface CRT observed in pre-apoptotic SKov3 and OVcar3 cells (*P* < 0.5). These findings indicated that treatment with TUDCA reduces detection of cell surface CRT under ER-stress conditions. Furthermore, treatment of OVcar3 or SKov3 cells with TUDCA significantly reduced the binding of extracellular FITC-CRT to cells treated with 2.5 μM DX irrespective of cell viability state’ (Fig. 4e, f).

### Effect of ER-stressors and inhibitors on CRT expression and release from OVcar3 cells

**Fig. 5:**
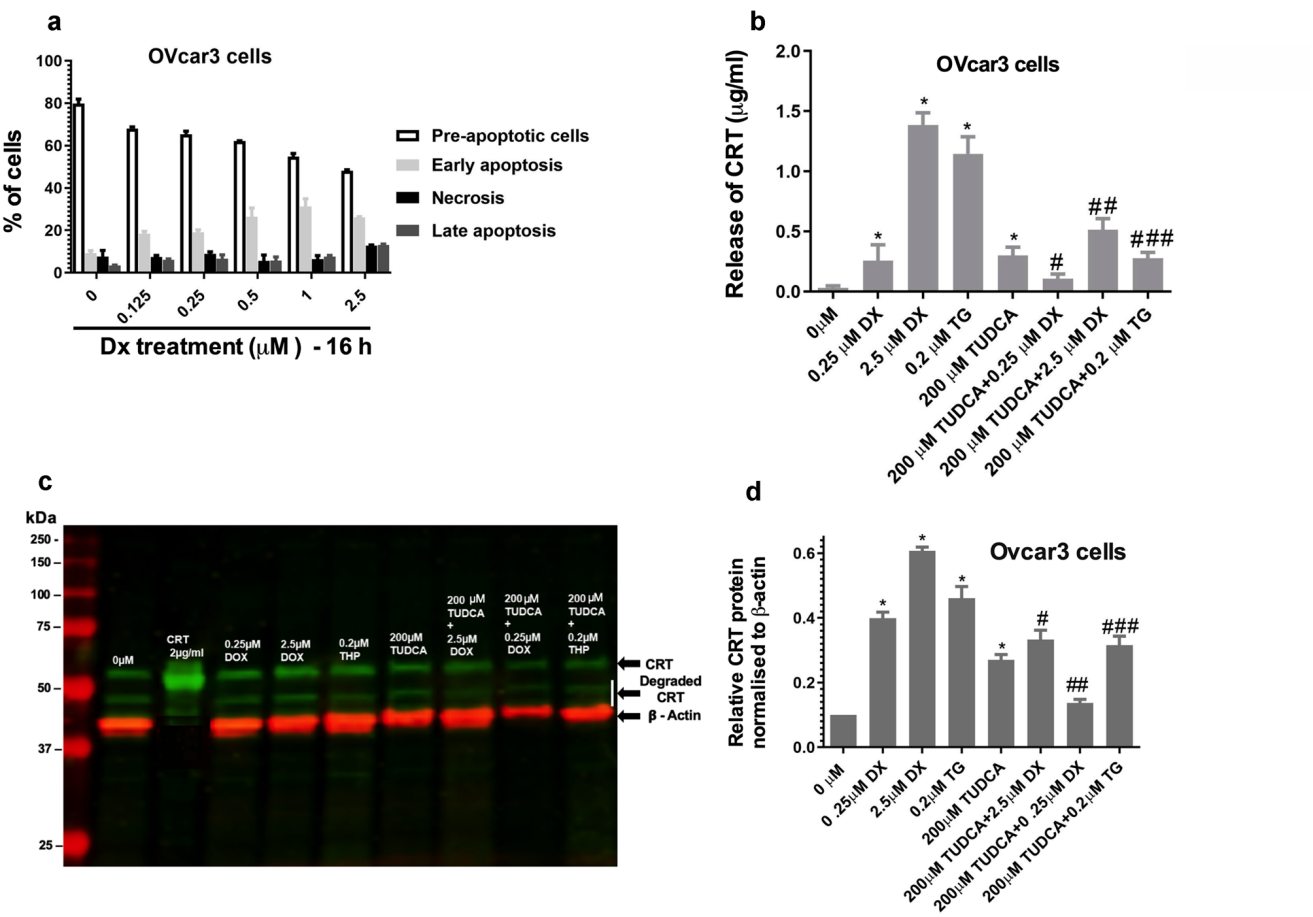
Effect of doxorubicin and thapsigargin on secretion and expression of CRT in OVcar3 cells. **a** Effect of a range of DX concentrations (0.125–2.5 µM) of OVcar3 viability. **b** ELISA of the mean ± SD concentrations of CRT released from OVcar3 cells after treatment with DX (0.2, 2.5 µM), TG (0.2 µM) for 16 h ± TUDCA. Student ‘t’ test was conducted on pooled data (results represent 6 independent experiments *P = 0.05 vs. control). **c** Representative western blot of CRT expression in OVcar3 cell lysates treated under the described conditions as above. CRT is depicted as green bands, actin as red bands. **d** Densitometry plots of CRT protein expression relative to β-actin as the loading control. Data are mean and SD of 10 independent experiments. *0 µM versus 0.25 µM DX, 0.2 µM DX, 0.2 µM TG, 200 µM TUDCA; ^#^2.5 DX versus 2.5 µM DX + 200 µM TUDCA, ^##^0.25 µM DX versus 0.25 µM DX + 200 µM TUDCA, ^###^0.2 TG µM versus 0.2 TG µM + 200 µM TUDCA. *P* = < 0.0200 for all

The over-expression of CRT has been observed in numerous tumour tissues and is considered a biomarker of elevated ER stress associated with malignancy [34], including ovarian cancer [29, 35, 36]. As an approach to investigate CRT expression and release from ovarian cancer cells under ER stress conditions, OVcar3 cells were treated with DX (0.125–2.5 µM), TG (0.2 µM) for 16 h ± 200 µM TUDCA. We examined the effect of this range of DX on OVcar3 cells viability. Approximately 70% of OVcar3 cells treated with low doses of DX (1.25–0.25 µM) remained in a pre-apoptotic state (Fig. 5a). After 16 h, the cells were removed from the cell culture medium for immunoblotting, and the CRT present in the cell-free media was quantified by ELISA (Fig. 5b). There was minimal base level of CRT released from untreated cells (~ 0.1 μg/ml), but interestingly, there was a significant increase in release of CRT (~ 0.3 μg/ml; *P* < 0.05) from the pre-apoptotic cells treated with 0.25 µM DX compared to untreated cells. As expected, cells treated with higher concentrations of DX and therefore containing a higher proportion of apoptotic and necrotic cells released greater quantities of CRT into the medium in a dose response manner (Fig. 5a, b). Similarly treatment of cells with 0.2 μM TG led to a significant release of CRT compared to untreated cells. The release of CRT into the medium was significantly reduced upon treatment of cells with DX or TG in the presence of TUDCA. CRT abundance was analysed in lysates prepared from OVcar3 cell treated as described above, with a typical blot shown (Fig. 5c). To semi-quantify the CRT abundance, several immunoblots were analysed by densitometry (Fig. 4d) of the pooled data (*n* = 10). Relative CRT abundance was highest in cells treated with DX or TG alone, and this was reduced in the presence of TUDCA. Both the cell free CRT ELISA data and cellular expression data agreed that increased ER-stress led to increased endogenous cellular CRT and subsequent release of the protein from stressed cells.

### The effect of CRT on maturation of THP-1-derived imDCs

Since ICD requires DC maturation, cancer cells undergoing ICD have CRT on their cell surface either as a result of endogenous CRT release (Fig. 3) or from CRT released from dead and dying cells (as shown in Fig. 5). Experiments were carried out to examine whether CRT alone can induce DC maturation. Immature DCs were incubated with increasing concentrations of CRT, and maturation was assessed by surface marker staining. The data presented in Fig. S7a, b demonstrate that exogenous CRT can induce maturation of THP-1 derived imDCs. This effect appears to be concentration dependent with 0.5 μg/ml CRT only inducing a significance increase in the expression level of HLA-DR, although it did induce a significant reduction in the expression level of CD14 from 69.72 ± 15.08 to 6.5 ± 2.30. In contrast, both 1 and 2.5 μg/ml CRT induced significant changes in the maturation surface markers CD14, CD11c, CD80, CD83 and HLA-DR. For instance, HLA-DR levels were increased from 58.73 ± 10.17 to 80.47 ± 3.82 at 1 μg/ml CRT and 77.92 ± 4.46 at 2.5 μg/ml CRT. However, although CD86 levels were increased by both 1 and 2.5 μg/ml CRT, these increases did not reach significance. CRT treatment also significantly reduced CD14 to 7.04 ± 2.628 at 1 μg/ml CRT, and 5.8 ± 2.775 at 2.5 μg/ml CRT, respectively, vs imDCs (69.72 ± 15.08).

## Discussion

CRT is an important intracellular chaperone protein normally located in the ER where is essential for glycoprotein folding and transport and calcium homeostasis [12, 37, 38]. However, an ever-increasing number of reports have observed extracellular release of CRT from numerous cell types where CRT takes on a variety of immunomodulatory roles [39]. The extracellular role of CRT in immunogenic cell death of tumour cells has been of interest in recent years [5, 40, 41]. As extracellular CRT on the surface of tumour cells appears to promote their recognition and elimination by the host immune system, further understanding of the mechanisms involved in CRT translocation from the ER to the cell surface is required. A consensus is that various chemical (anthracyclines, fatty acids) and physical (radiation) perturbations of tumour cells can lead to redistribution of CRT from the ER to the cell surface [11, 42]. In addition, both specific protein–CRT interactions and cell death pathways have been proposed to promote CRT translocation to the cell surface [34, 41, 43, 44]. This has led to the proposal that inducing selective CRT surface expression on tumour cells may be a therapeutic means of targeting cancer cells for eradication by cell mediated immunity [5, 9, 18, 40]. There does not appear to be a common mechanism that can lead to the endogenous release of CRT to the cell surface. Previously it has been shown that agents that reduce ER calcium levels, e.g., TG induces cell surface expression of CRT on pre-apoptotic mouse embryonic fibroblasts (7-Aminoactinomycin D-7 AAD-/AnnV-) [45, 46] in a CRT-ERp57 independent manner.

Using several human ovarian cancer cell lines, we have shown here that TG [47], as well as DX induces CRT translocation from the ER to the cell surface on these cancer cells. Both TG and DX are known to induce UPR, an ER stress coping response, but by different modes of action. TG inhibits calcium pumps leading to depletion of Ca^2+^ stores in the ER. DX in contrast is an anti-cancer drug that induces UPR/ER stress [48] and apoptosis by intercalating into a number of key DNA and RNA polymerases and altering the Bcl-2/Bax central checkpoint apoptosis pathways which are prevalent in ovarian cancer cells [49]. A side effect of DX in the treatment of cancer patients has helped us understand its role in ER stress. DX is particularly toxic to cardiomyocytes and can directly lead to dilation of the ER, resulting in an increase in the volume of the ER lumin that is considered an ultrastructural signature of ER stress [50]. Fu and Coworkers have proposed that DX induces the ER stress by activating the ER transmembrane protein ATF6 and simultaneously decreasing XBP1 expression which results in reduced induction of the ER stress inhibitor GRP78 [51]. However, it is unknown if this modality occurs in cancer cells. Interestingly, when GRP78 expression remains high as observed in some breast cancer specimens, a shorter recurrence-free survival occurs in patients who receive DX [52]. We demonstrated that TG and DX induced endogenous CRT to translocate to the cell surface and into the extracellular environment. Release of CRT and other DAMPs into the extracellular environment provides further opportunity for the released CRT to bind to proximal cancer cells making them susceptible to ICD. Interestingly others have suggested immune cells such as macrophages are capable of releasing CRT functioning to detecting cancer cells through trans interaction with as yet unidentified specific receptors on target cancer cells [53]. Independent studies have identified a number of the unfolded protein response (UPR) pathway components associated with ER stress (e.g. PERK and eIF2α) influence CRT production upon TG and DX treatment of cancer cells [43, 54]. Combined these events implicate DX in the induction of both ER stress and UPR at least in some cell types.

We observed that the amount of CRT present on the cell surface of ovarian cancer cells or released into the extracellular milieu was dependent on the cell viability status. Higher levels of CRT were released from cells when in a late apoptotic or necrotic state compared to early apoptosis or living cells (Fig. 1). The viability status of the cells also influenced the ability of extracellular-CRT to bind to cells (Fig. 2). Previously we showed that CRT binds directly to phosphatidylserine (PS) [44], and since both early and late apoptotic cells have increased external membrane exposed PS as assessed by AnnV binding, it follows that CRT may bind to the cell surface via available PS. However, it has been shown previously that CRT appears on the surface of mouse colon cancer cells treated with DX, before increased surface PS is evident, generating an ‘eat-me’ signal that promotes phagocytosis of tumour cells and the presentation of the resulting antigens by dendritic cells [5, 10]. In our study, we also observed increased levels CRT on the surface of pre-apoptotic (PS surface negative) SKov3 cells treated with DX compared to untreated cells (Fig. 3) which is in agreement with others employing murine cancer cells [5]. Since CRT binds to AnnV^−ve^ pre-apoptotic cells, it is unlikely under these conditions for CRT to be binding to PS already bound to AnnV. As well as PS, we and others have shown CRT can bind to other cell surface proteins, for example, thrombospondin-1 ((TSP1), that upon binding to CRT regulates integrin-dependent cell adhesion [55, 56]. Many tumour cells express TSP1 on their cell surface, and it has been proposed to affect cancer cell immunity and adhesion. TSP-1 is known to prevent DX-induced apoptosis in some human cancer cells [57]. One might speculate that CRT-TSP1 interaction may affect the apoptosis process. As well as TSP-1, a number of alternative CRT binding proteins have been proposed on the surface of cancer cells.. These include integrins [58], fibrinogen [59], laminin [60] surface ERP57 [61]. Paradoxically, despite the immunogenic properties of cell surface CRT, many malignant neoplasms express higher intracellular CRT as a cell survival factor, possibly allowing tumour cells to adapt to rapid changes in cellular metabolism and calcium requirements during rapid protein production and cellular proliferation and metastases [62]. Thus, cancer cells expressing increased levels of intracellular CRT, potentially have an elevated amount of CRT that may be release exogenously via the stress induced by DX and TG treatments and could possibly bind to a number of surface proteins and increase their immunogenicity, Our results show treatment of cells with TG and DX led to an increase in CRT protein detection and release of CRT from cells compared to untreated cells (Fig. 5b).

The treatment of cancer cells with DX and/or TG-cell treatment led to CRT release that could be inhibited by an ER-stress inhibitor TUDCA. Our data show that TUDCA effectively alleviates the ER stress triggered by TG and DX as indicated by a decrease in cell surface CRT and increase in cell survival (Fig. 5). ER stress is known to be a factor involved in alteration of normal ovarian tissue morphology and inducer of cancer initiation [63]. This adds further evidence that activation of UPR mediator ER stress acts as a driver of CRT release from various human ovarian cancer cells. Recently, a retrospective clinical study of epithelial tumour cells from high-grade serious carcinoma (HGSC) patients who had not received neoadjuvant chemotherapy was examined for correlation between ER stress markers (BIP, CHOP and Hsp70) and CRT expression. The study noted that there was a positive correlation in cells with high levels of ER stress biomarkers and CRT expression, but patients with low CRT expression correlated with advanced disease. Overall the study suggested doxorubicin-induced ER stress might be an appropriate adjuvant treatment for ovarian cancer patients [20].

In this study, we demonstrated extracellular FITC-CRT also bound to cancer cells. These experiments demonstrate that irrespective of the type of mechanism inducing CRT cell surface exposure or release from dead or dying cells, once on the cell surface, CRT can act as a “damage-associated molecular pattern” (DAMP), potentially enhancing the uptake of tumour cells by professional phagocytes such as DCs and resulting in a T-cell mediated tumouricidal response. To test this hypothesis in part, we investigated whether extracellular CRT could promote maturation of imDCs from THP-1 cells. CRT at concentrations we saw released from cancer cells significantly increased the levels of the DC surface markers CD11c, CD86 and HLA-DR, although the increase in levels of CD80 and CD83 did not reach significance. These data agree with similar reports in the literature. For instance, it has been reported that THP-1 cells and the CD34 + (KG-1) leukaemia cell line incubated with varying concentrations of rhGM-CSF and rhIL-4 for 5 days showed upregulated levels of CD11c, CD80 and CD86 and of the cell-surface receptors CD40, CD209 (DC-SIGN) but failed to express CD83 [64]. What is not clear is how CRT induces a DC response. In a recent study, we have shown that CRT interacts with pathogen associated molecular patterns (PAMPs), specially lipopolysaccharide (LPS). The physical binding interaction of CRT with LPS suggests a role for this interaction in DAMP-dependent PAMP immunity such as, enhancing innate and adaptive immunity [65]. Consequently, extracellular CRT absorbed onto the surface of tumour cells or endogenous CRT released from the cells may act as a ‘sponge’ for low levels of LPS that in turn elicits an immune response. A recent study showed that the release of CRT from dying cells, prior to PS cell surface appearance, binds to macrophages, thereby inducing numerous immune activities including pro-inflammatory cytokines, cell polarization and migration [66]. Other have demonstrated that CRT and other chaperones elicited immune functions against tumours in vivo possibly due to LPS binding and showed that following removal of associated LPS, numerous immune responses were lost except ERK phosphorylation [67].

## Conclusion

It is believed that immunogenic cell death of tumour cells is triggered when CRT is translocated to the cell surface of tumour cells where it acts as an ‘eat-me’ signal. Here we show that a range of concentrations of DX and/or TG as ER stressors enhance release of endogenous CRT from human ovarian cancer cells. In addition, both endogenously released and exogenous CRT binds to cancer cells. However, the state of the cell’s viability correlates with the ability of CRT to bind to the cells. Extracellular CRT can also bind to a lesser extent directly to untreated tumour cells and pre-apoptotic cells. Under ER stress conditions induced by DX or TG-treatment, CRT binds predominantly to ovarian cancer cells in a state of early-late apoptosis and necrosis. Both the release and binding of CRT to ovarian cancer cells can be inhibited upon treatment with an ER stress inhibitor and proteostasis promoter (TUDCA). These findings confirm that ER-stress regulates in part the release and binding of CRT to cancer cells where it may play a role in ICD. This possibility is currently under investigation in our lab.

## Supplementary Information

Below is the link to the electronic supplementary material.

Supplementary file1 (PDF 1765 kb)
