# Supplementary material for: Endoplasmic reticulum stress-induced release and binding of calreticulin from human ovarian cancer cells
Source: Cancer Immunol Immunother. 2021 Nov 20;71(7):1655–69. doi: 10.1007/s00262-021-03072-6 (PMC9188521; doi:10.1007/s00262-021-03072-6)
Supplement: Supplementary file 1 — Supplementary file1 (PDF 1765 kb) [file 262_2021_3072_MOESM1_ESM.pdf]

Appendix A. Supplementary Data

Fig.1 S1 Representative plot of cell viability of SKov3 ovarian cancer cells.

Fig. S2 Evaluation of free FITC and FITC-CRT or non-specific FITC-IgG binding to OVcar3 cells.

Fig. S3 CRT ELISA standard curve.

Fig. S4 Morphological characteristics of the cancer cell lines studies and their binding to FITC-labelled CRT or non-specific FITC-IgG.

Fig. S5 The effect of doxorubicin (DX) and thapsigargin (TG) alone and in combination on OVcar3 and SKov3 ovarian cancer cell viability.

Fig. S6 Cy5 secondary antibody alone does not bind to SKov3 cells treated with doxorubicin.

Fig. S7 Extracellular CRT induces expression of mature dendritic cell (mDC) phenotypic markers in THP-1 derived immature dendritic cells (imDCs).

Table S1 Details of the antibodies and isotype controls used for characterization of imDCs.

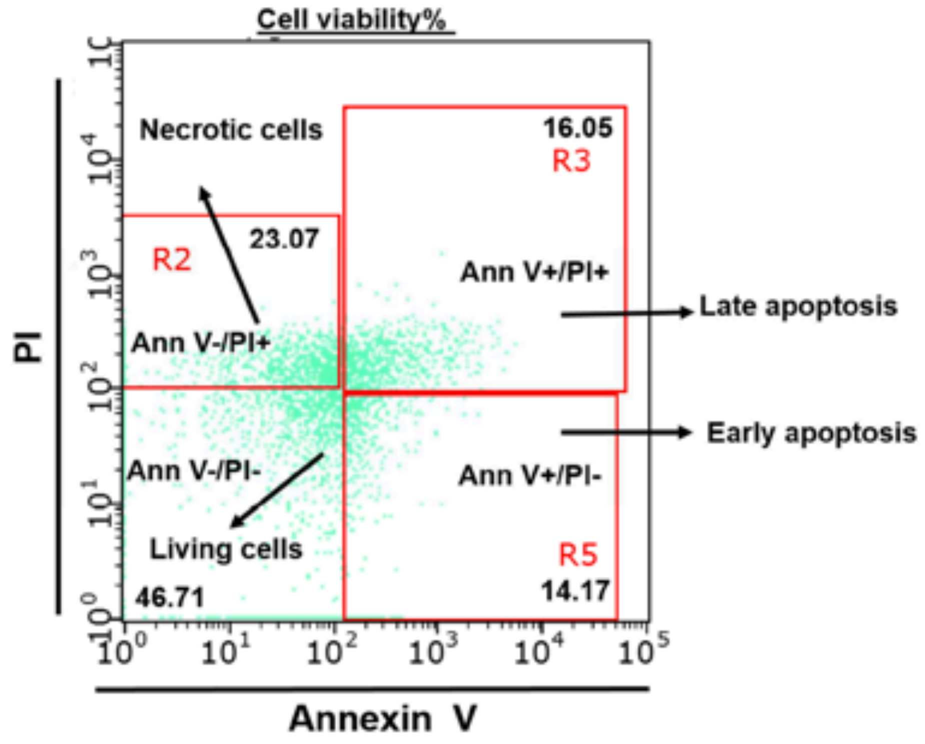

Fig.1 S1 Representative plot of cell viability of SKov3 ovarian cancer cells. Cell viability was assessed by flow cytometry following dual staining with annexin V (Ann V) and propidium iodide (PI). Living (pre-apoptotic) cells are located in bottom left quadrangle.

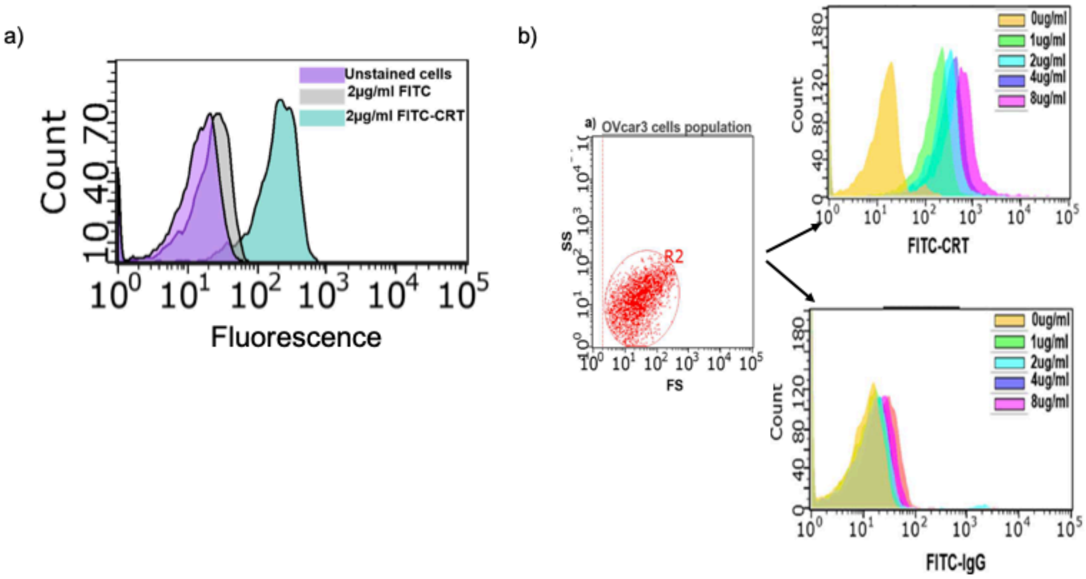

Fig. S2 Evaluation of free FITC and FITC-CRT or non-specific FITC-IgG binding to OVcar3 cells. a. Ovcar3 cells were incubated with free 2 μg/ml FITC or 2 μg/ml FITC-CRT for 30 min in the dark and then excess probes washed off. The remaining fluorescent intensity of FITC and FITC-CRT bound to cells was determined by flow cytometry. Free FITC does not bind to the surface of OVcar3 cancer cells. **b.** FITC-CRT and FITC-IgG binding to Ovcar3 cells treated with different concentrations (1 μg – 8 μg/ml) of FITC-labelled protein or untreated (autofluorescence control) and analysed by flow cytometry.

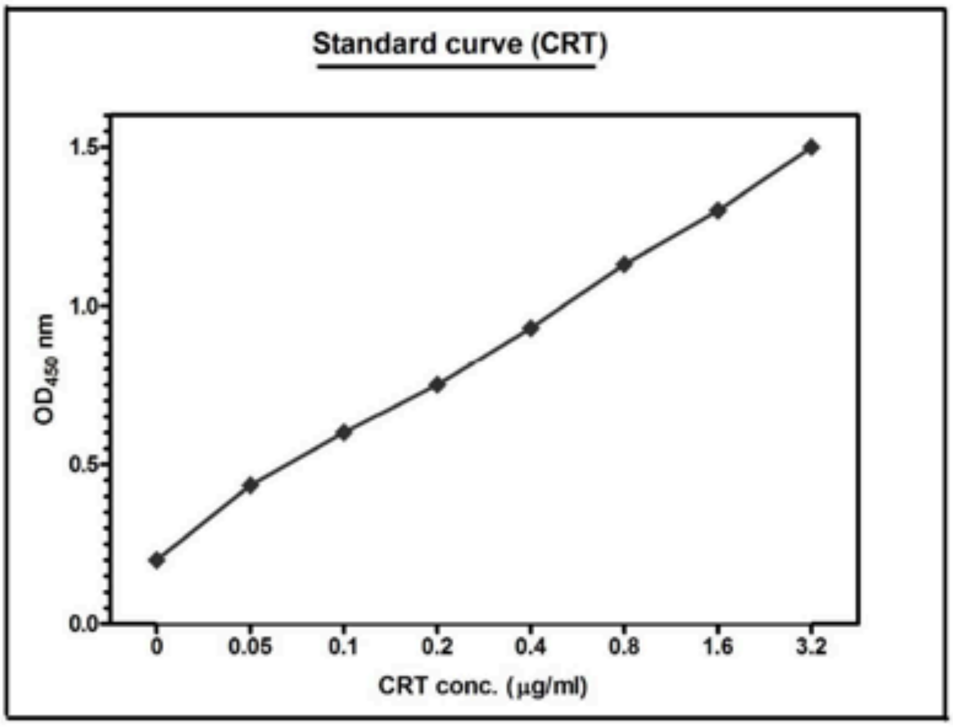

Fig. S3 CRT ELISA standard curve. Known concentrations of CRT (0.05 µg – 3.2 µg/ml) diluted with binding buffer were plated onto wells of an ELISA plate according to the manufacturers instructions. A standard curve was constructed by plotting CRT concentrations vs. OD450 nm to provide a reference calculation of CRT levels released from cancer cells after various treatments.

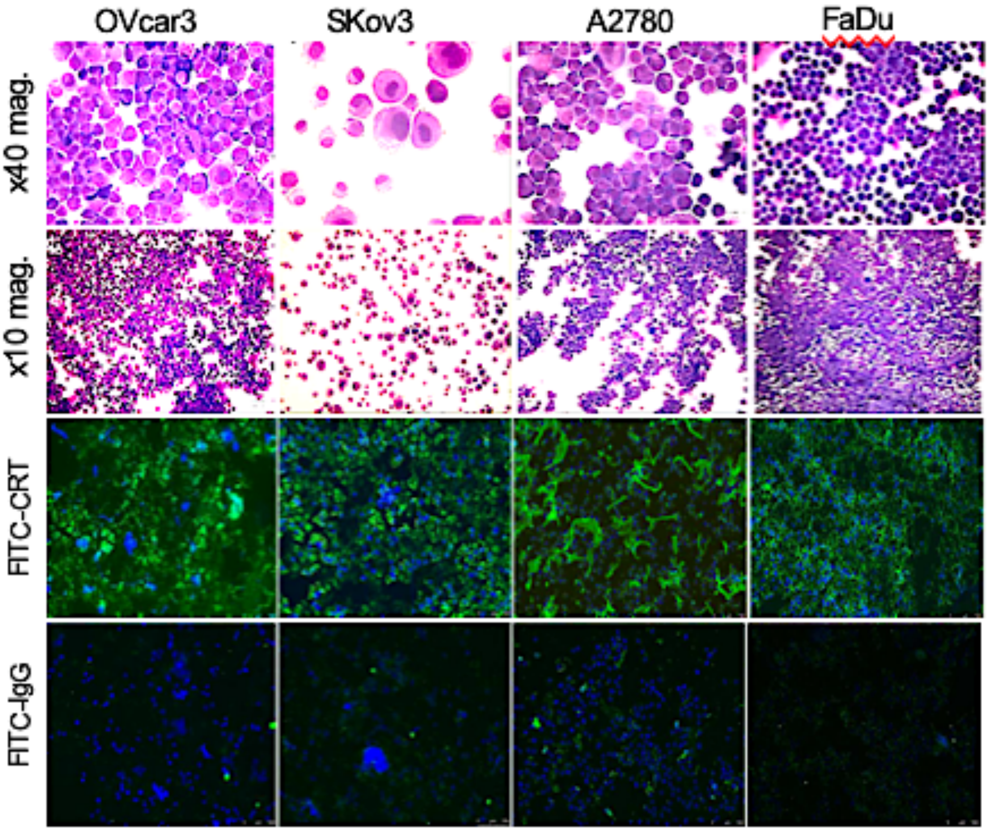

Fig. S4 Morphological characteristics of the cancer cell lines studies and their binding to FITC-labelled CRT or non-specific FITC-IgG. Cultured cells were stained with Diff-Quick and observed by light microscopy at 40x magnification (row one), 10x magnification (row two). Differences in 2 μg/ml FITC-CRT (row three) and 2 μg/ml FITC-IgG (row four) binding to the surface of cancer cells (green), as assessed by fluorescence microscopy at 40x magnification. Cell nuclei are stained with DAPI (blue). OVcar3 (column 1); SKov3 (column 2); A2780 (column 3); FaDu (column 4).

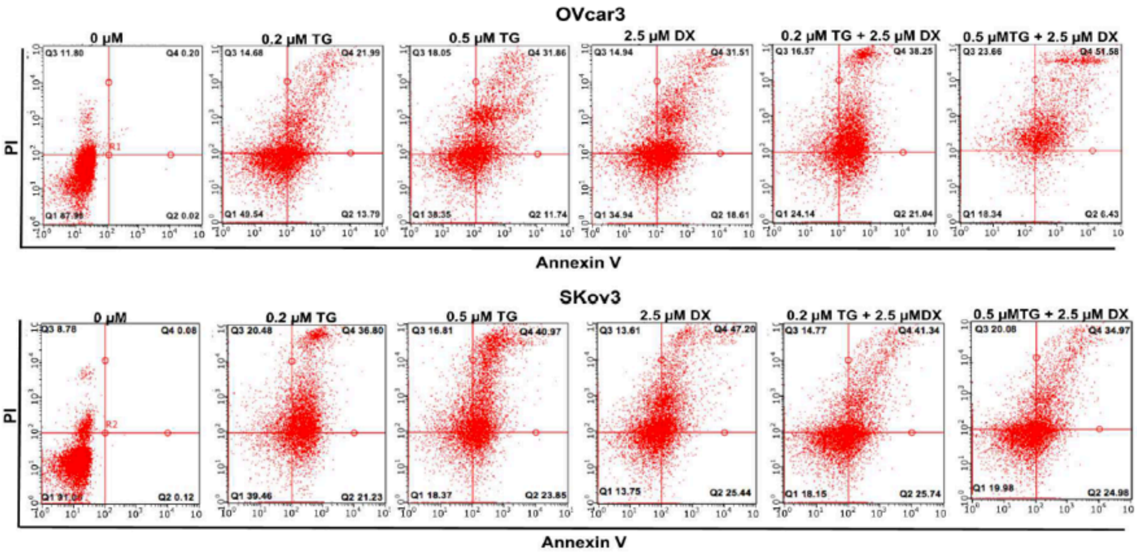

Fig. S5 The effect of doxorubicin (DX) and thapsigargin (TG) alone and in combination on OVcar3 and SKov3 ovarian cancer cell viability. Cells were treated with DX (2.5 μM) ± TG (0.2, 0.5 μM) for 16 h and their apoptosis/necrosis status was determined by flow cytometry using annexin V and propidium iodide (PI) staining before and after treatment.

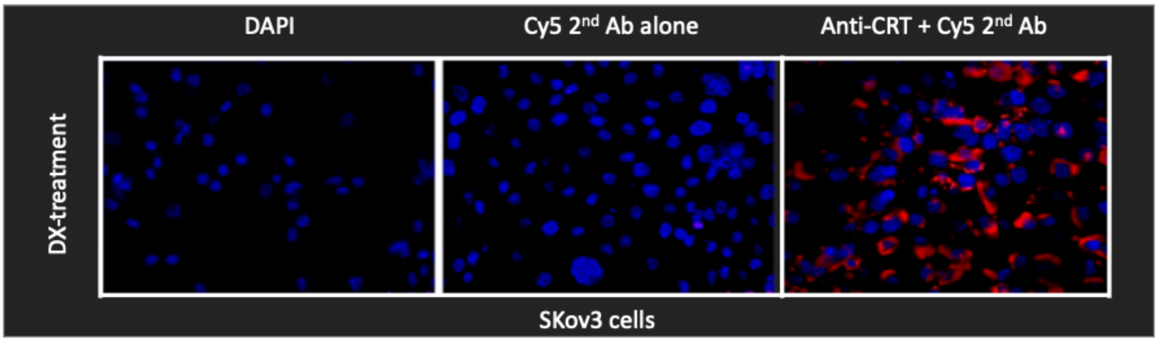

Fig. S6 Cy5 secondary antibody alone does not bind to SKov3 cells treated with doxorubicin. SKov3 cells were treated ± 2.5 μM DX for 16 h and then incubated with Cy5-conjugated secondary antibody alone (Abcam- 6564; 12000 dil.) or anti-human CRT (Thermofisher A3-900; 1:200 dil.) + 2nd ab for CRT by immunocytochemistry.

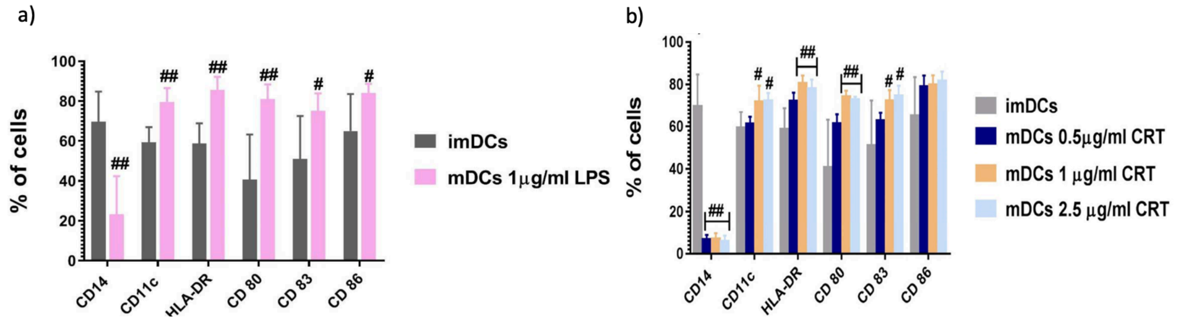

Fig. S7 Extracellular CRT induces expression of mature dendritic cell (mDC) phenotypic markers in THP-1 derived immature dendritic cells (imDCs). a. imDCs derived from THP-1 cells were matured by incubation with lipopolysaccharide (LPS) for 4 days and the change in surface levels of CD11c, CD80, CD83, CD86 and HLA-DR and CD14 were measured by flow cytometry. Cells were stained for each marker before (imDC) and after (mDC) LPS treatment. N = 5, # $p<0.05$, ## $p<0.01$ for significant difference between no. of imDcs vs. mDcs cells displaying various maturation markers. **b.** THP-1 derived imDCs were stimulated with different concentrations of CRT for 4 days. DC maturation was assessed by flow cytometric analysis of mDC surface markers. Cells were treated with between 0.5 μg/ml & 2.5 μg/ml CRT. Data are shown as mean ± SD. N = 5, # $p<0.05$, ## $p<0.01$ for significant difference in number of cells at 7 days maturation with and without CRT treatment.

Markers	Dilution factors	Isotype controls	Dilution factors
Alexa Fluor® 488 anti-human CD14 Antibody	1:50	Alexa Fluor® 488 Mouse IgG1, κ Isotype Ctrl (FC) Antibody	1:20
PE anti-human CD11c Antibody	1:50	PE Mouse IgG1, κ Isotype Ctrl (FC) Antibody	1:20
PE anti-human CD80 Antibody	1:50	PE Mouse IgG1, κ Isotype Ctrl (FC) Antibody	1:20
PE anti-human CD83 Antibody	1:25	PE Mouse IgG1, κ Isotype Ctrl (FC) Antibody	1:20
PE anti-human CD86 Antibody	1:50	PE Mouse IgG1, κ Isotype Ctrl (FC) Antibody	1:20
PE anti-human HLA-DR Antibody	1:100	PE Mouse IgG2b, κ Isotype Ctrl Antibody	1:11

Table S1 Details of the antibodies and isotype controls used for characterization of imDCs.
